# Ketone Bodies and Cardiovascular Disease: An Alternate Fuel Source to the Rescue

**DOI:** 10.3390/ijms24043534

**Published:** 2023-02-10

**Authors:** Antonis S. Manolis, Theodora A. Manolis, Antonis A. Manolis

**Affiliations:** 1 School of Medicine, Athens University, 115 27 Athens, Greece; 2 School of Medicine, Patras University, 265 04 Patras, Greece

**Keywords:** ketone bodies, beta-hydroxybutyrate, acetoacetate, acetone, cardiovascular disease, heart failure, myocardial infarction, fatty acid oxidation, cardiac metabolism, cardiac energetics, adenosine triphosphate

## Abstract

The increased metabolic activity of the heart as a pump involves a high demand of mitochondrial adenosine triphosphate (ATP) production for its mechanical and electrical activities accomplished mainly via oxidative phosphorylation, supplying up to 95% of the necessary ATP production, with the rest attained by substrate-level phosphorylation in glycolysis. In the normal human heart, fatty acids provide the principal fuel (40–70%) for ATP generation, followed mainly by glucose (20–30%), and to a lesser degree (<5%) by other substrates (lactate, ketones, pyruvate and amino acids). Although ketones contribute 4–15% under normal situations, the rate of glucose use is drastically diminished in the hypertrophied and failing heart which switches to ketone bodies as an alternate fuel which are oxidized in lieu of glucose, and if adequately abundant, they reduce myocardial fat delivery and usage. Increasing cardiac ketone body oxidation appears beneficial in the context of heart failure (HF) and other pathological cardiovascular (CV) conditions. Also, an enhanced expression of genes crucial for ketone break down facilitates fat or ketone usage which averts or slows down HF, potentially by avoiding the use of glucose-derived carbon needed for anabolic processes. These issues of ketone body utilization in HF and other CV diseases are herein reviewed and pictorially illustrated.

## 1. Introduction

Cardiac metabolism, which is highly active, involves the oxidation of fatty acids as the main source (40–70%) for generating energy via the synthesis of adenosine triphosphate (ATP) [1,2]. While glucose and glycolysis provide substrates to about 20–30% of basal myocardial metabolism, other alternate substrates, comprising amino acids, lactate and ketone bodies, furnish modest quantities (5–20%) to basal ATP generation in the normal heart in adults [2]. 

The alterations in myocardial metabolism, particularly the fatty acid β-oxidation taking place in the mitochondria and resulting in the generation of ketone bodies (ketogenesis), have always been a main focus of research in the study of the emergence and progression of heart failure (HF) and other cardiovascular (CV) diseases (CVD) [3,4]. During situations that lead to exhausted glucose levels in the circulation, ketone bodies produced in the liver can constitute fuel substrates for the brain, while current evidence indicates that the heart, which utilizes mainly fatty acids as an alternative fuel source, can also avail itself from ketone bodies as a fuel source [4]. Ketone body metabolism preserves bioenergetic homeostasis when dietary carbohydrates are limited. Ketone bodies control mitochondrial metabolism, the generation of reactive oxygen species (ROS), and energetics through their oxidation and may thus have important signaling tasks within myocardial cells. The metabolism of ketone bodies may impact several human disease conditions pertinent to CV diseases, comprising diabetes, obesity, atherosclerosis, and HF. Furthermore, enzymes participating in ketone metabolism may be subject to pharmacological manipulation. However, first, one needs to disclose the mechanisms via which ketone body metabolism affects CV pathophysiological functions and processes [4]. 

Ketone bodies comprise a family of three small low-molecular-weight hydrosoluble molecules, acetoacetate (AcAc), beta-hydroxybutyrate (β-OHB) (also known as 3-hydroxybutyrate), and acetone, which are synthesized mainly in the hepatic mitochondria and constitute a form of alternate fat-derived metabolic energy source for vital organs, including the heart, in states of nutrient shortage when glucose availability is low [5,6]. Ketone bodies are low-chain organic molecules with four (AcAc and β-OHB) or three carbon atoms (acetone), with β-OHB and acetone being the principal circulating ketone bodies; β-OHB accounts for ~70% of the total circulating ketones [7]. It seems that extrahepatic tissue ketone concentrations do not exceed that in circulation [7]. In normal conditions, ketone body concentrations are low; however, under situations of oxidative stress (e.g., fasting, exercise, acute illness), their concentrations are elevated [8]. Interestingly, in patients with HF, ketone body plasma levels are also increased. This increase is analogous to the degree of cardiac dysfunction and neurohormonal activation, partly ascribable to increased free fatty acid mobilization in response to heightened neurohormonal stimulation [9]. A positive correlation has been noted between the enhanced energy metabolism of cardiomyocytes and the concentrations of β-OHB and acetone. Importantly, it seems that the mild ketosis created by sodium-glucose cotransporter 2 (SGLT-2) inhibitors is one of the potential pathophysiologic mechanisms accounting for the important CV and renal advantages noted in patients with type 2 diabetes (T2D) who are receiving these drugs [10]. 

## 2. Ketogenesis/Ketolysis 

In the liver, free fatty acids (FFAs) go into the cytoplasm of liver cells and then into the mitochondria, aided by fatty acid transporters and carnitine palmitoyltransferase I, which act as substrates for ketogenesis, whereby the ketone bodies, β-OHB, AcAc, and acetone are produced. Thus, ketogenesis involves a metabolic pathway that produces ketone bodies, mainly in the liver [11,12], a process that starts with FFAs which are subsequently converted into acetyl-coenzyme A (CoA) through mitochondrial β-oxidation, which is the access point into the cycle of Krebs [3]. This occurs when lipolysis predominates over lipogenesis with a resultant rise in FFAs. Alternatively, and during conditions of carbohydrate shortage or unavailability, acetyl-CoA functions as a ketogenic substrate. In the mitochondrial matrix, acetyl-CoA is condensed with acetoacetyl-CoA via 3-methylglutaryl-CoA (HMGC) synthase 2 to produce HMGC and is subsequently converted by HMGC lyase to acetoacetate. Acetoacetate is then reduced by D-b-hydroxybutyrate dehydrogenase (BDH1) to produce β-OHB, which is channeled into the bloodstream by the solute carrier 16A family on liver cells [13,14,15]. Ketone bodies that are synthesized in the liver are then excreted and exported from the liver cells via the monocarboxylate transporters (MCTs) 1, 2, and 7 and enter the circulation. 

**Figure 1:**
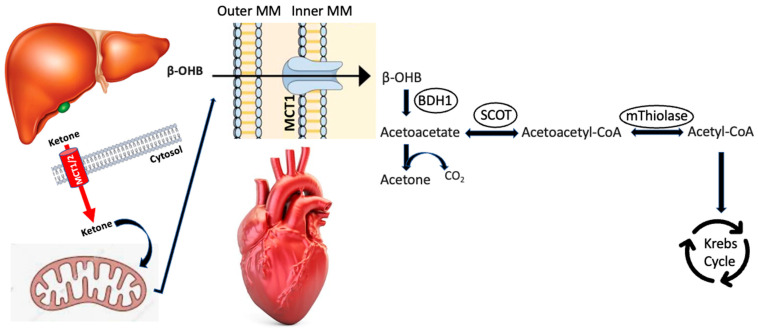
The schema illustrates ketone body production and oxidation. Ketone bodies produced in the liver are released via the monocarboxylate transporter (MCT) 1/2 into the circulation and reach extrahepatic tissues, including the heart, where they can be utilized as energy sources. Ketone body oxidation in cardiac tissue involves beta-hydroxybutyrate dehydrogenase 1 (BDH1), bound to the inner mitochondrial membrane (MM), which converts beta-hydroxybutyrate (β-OHB) to acetoacetate. Acetoacetate can then be activated via succinyl-CoA:3-oxoacid-CoA transferase (SCOT) whereby a succinyl CoA is transferred to acetoacetate in order to form acetoacetyl CoA. Acetoacetyl CoA is subsequently cleaved to yield two acetyl CoA molecules which can then be oxidized in the tricarboxylic acid (Krebs) cycle. BDH1 = beta-hydroxybutyrate dehydrogenase; βOHB = beta-hydroxybutyrate; CoA = coenzyme A; CO2 = carbon dioxide; MCT = monocarboxylate transporter; MM = mitochondrial membrane; mThiolase = mitochondrial thiolase; NADH = nicotinamide adenine dinucleotide reduced; SCOT = succinyl-CoA:3-oxoacid-CoA transferase.

In the heart, circulating ketone bodies get access to the cardiomyocyte mitochondria via MCTs 1 and 2 and act as substrates for ketolysis, finally producing acetyl-CoA which moves into the cycle of Krebs (Figure 1) [14,15]. During ketolysis, β-OHB which has entered the myocyte mitochondria via these two transporters (MCT1/2) is converted back to acetoacetate by BDH1 [16], then transformed into acetoacetyl-CoA by succinyl-CoA, 3-ketoacid-CoA transferase/3-oxoacid CoA-transferase 1 (SCOT/OXCT1), and in the end by conversion of mitochondrial acetoacetyl-CoA thiolase into two acetyl-CoA molecules that enter the tricarboxylic acid (Kreb’s) cycle to generate ATP [7]. 

Thus, the heart utilizes both β-OHB and acetoacetate, with the ratio of β-OHB to acetoacetate reflecting the mitochondrial nicotinamide adenine dinucleotide hydrogen (NADH) to nicotinamide adenine dinucleotide (NAD) redox ratio [17,18]. Utilization of acetoacetate is enhanced in diabetic animal hearts, as well [19], which can improve systolic function through an anti-oxidation mechanism [20]. Relying on this finding, acetoacetate may qualify as a prognostic biomarker for HF, whereby elevated concentrations of acetoacetate confer a higher death rate in patients with HF [21], which contradicts its prior purported protective effects. This may be justified by varying disease states, comprising factors such as the degree of HF and incident diabetes mellitus (DM) [21]. Furthermore, in contrast to β-OHB, alterations in acetoacetate concentrations are less influenced by stress, indicating that β-OHB is a more stress-responsive ketone body [22]. 

Summarizing, ketone bodies, β-OHB, and acetoacetate, synthesized in the liver, are excreted by the liver cell through the MCT 1 and, via the circulation, arrive at extrahepatic tissues, including the heart, where they can be utilized as energy fuel [6]. Peripherally, β-OHB is reconverted into acetoacetate and then, as said, into acetoacetyl-CoA, which gets access into the Krebs cycle. A small fraction of acetoacetate is automatically decarboxylated into acetone that is mostly exhaled via the respiratory system [7]. When circulating ketone body levels are within normal limits, they serve as energy substrates for various organs including the heart, muscles, kidney, brain, and mammary glands. It is observed that red blood cells, which do not contain mitochondria, cannot use ketone bodies as substrates for energy. In the same way, the liver, which does not contain the enzyme Oxct1/SCOT1 to metabolize acetoacetyl-CoA, can generate but not break down ketone bodies. As indicated, ketone bodies are an alternative fuel under situations of carbohydrate shortage such as fasting for long durations or vigorous physical exertion. The metabolic turn towards ketogenesis is controlled by glucagon. Importantly, in contrast to other metabolic pathways, such as glycolysis or the catabolism of fatty acids (lipolysis) via β-oxidation that demand the use of energy in certain intermediate steps, the oxidation of ketone bodies is completely independent of ATP supply [7]. 

Exogenous infusion of acetoacetate has been shown to inhibit the utilization of FFAs by the heart and results in an increase in the respiratory quotient of the heart [23]. Exposure of myocardial cells to ketone bodies diminishes insulin sensitivity and systolic cardiac function [24]. 

Importantly, β-OHB may directly enhance transcriptional control via epigenetic modulation, and, thus, regulate or modify inflammatory processes. By reducing inflammasome, ketone bodies may contribute to the amelioration of insulin resistance and relative metabolic diseases. Furthermore, ketone bodies are anticipated to have an interaction with sirtuin-1 (SIRT1) (epigenetic interaction), thus initiating antioxidant pathways, and therefore have principal, synergistic roles (epigenetic regulators) for metabolic well-being [5]. 

Interestingly, recent data indicated that impaired ketogenesis may be a possible perplexing factor in advanced COVID-19 infection, as β-OHB serves as an alternate source of carbon that enhances T-cell responses in viral infections of the lung [25]. Indeed, a study in mice showed that a ketogenic diet and the supply of β-OHB as a ketone ester drink restored CD4^+^ T cell metabolism and function in severe lung infections, finally decreasing the death rate of mice afflicted by SARS-CoV-2 [25].

## 3. Ketone Bodies in Cardiovascular Disease 

As mentioned, ketone bodies are synthesized in the liver from acetyl-CoA that is mostly produced from fatty acid β-oxidation, taking place in the mitochondria [26]. During physiological conditions of reduced availability of carbohydrates and an excess of fatty acids, ketone bodies are transferred to tissues outside the liver for final oxidation [4,27]. The physiological conditions that ordinarily use ketone body oxidation comprise the neonatal period, post-exercise, starvation, and periods of low-carbohydrate diets [26,28,29]. Besides energy metabolism, ketone bodies act as substrates to lipid and sterol biosynthesis in many tissues, such as the liver, the developing brain, and the lactating mammary glands [26,30]. 

Targeting myocardial substrates (fatty acid, glucose, or ketone metabolism) might provide clinical benefits to patients with heart failure (HF) [31]. Due to high energy demands, the myocardium can avail itself of the benefits of augmenting ketone oxidation to preserve sufficient ATP generation. The adult heart utilizes fatty acids as a principal fuel source, but it can also get energy from other substrates such as glucose and ketone, and to some degree from lactate, pyruvate, and amino acids. Nevertheless, myocardial cells of the failing heart can withstand incredible metabolic remodeling that includes a change in substrate usage and decreased ATP production, which explain cardiac remodeling and abnormal systolic function. The principal therapeutic metabolic strategies comprise blocking fatty acid uptake/fatty acid oxidation, decreasing circulating fatty acid concentrations, augmenting glucose oxidation, and enhancing ketone oxidation.

During fasting, starvation, or a low-carbohydrate diet, the insulin concentrations in circulation are reduced, and this enhances lipolysis, and the catabolism of fat ends up as the principal energy source [32]. The energy metabolism of the liver is adjusted so that under these conditions, ketone bodies are produced from the β-oxidation of fatty acids and constitute an auxiliary fuel source, besides gluconeogenesis. Thus, high blood concentrations of ketone bodies suggest a dietary insufficiency of carbohydrates. 

The normal reference range for blood ketones is <1 mg/dL or <0.1 mmol/L in a fed state, and <6 mmol/L in a prolonged fasted state; levels of 0.6–1.5 mmol/L incur a slightly increased risk of diabetic ketoacidosis-DKA (there is a need for retesting in 2 h); levels of 1.6–2.9 mmol/L confer an increased risk of DKA and >3 mmol/L, very high risk of DKA (in need for medical assistance) (https://www.nhs.uk/conditions/diabetic-ketoacidosis/) (accessed on 11 January 2023) [8]. Most investigators define normal serum levels of ketone bodies as <0.5 mmol/L, hyperketonemia as levels >1.0 mmol/L, and ketoacidosis as levels >3.0 mmol/L [22]. Again, one should be cognizant of the fact that fasting raises ketone body levels which may be beneficial for patients with HF, as long as they do not have known or undiagnosed diabetes, in which case the grave risk of DKA is lurking. 

A recent animal study indicated that the endothelial cells of the heart can oxidize ketone bodies and that this promotes cell proliferation, migration, and vessel sprouting [33]. This oxidation mechanism and process needs succinyl-CoA:3-oxoacid-CoA transferase, a crucial enzyme of ketone body oxidation. An increase in ketone body concentrations by a high-fat, low-carbohydrate ketogenic diet has been shown to increase endothelial cell proliferation in mouse hearts. Interestingly, in a mouse model of cardiac hypertrophy, the ketogenic diet averted blood vessel rarefication, suggesting a possible favorable effect of dietary modification in CV diseases.

### 3.1. Ketogenic Diet 

With the development of HF, the heart uses ketone bodies at progressively higher percentages, suggesting an adjustable reaction to stress. Therefore, as more ketone bodies become available, protection against HF is enhanced. However, although a ketogenic diet is a widely employed approach to increase ketone body availability, cardioprotection afforded by a ketogenic diet against HF has not been established. An animal study investigating the influence of the ketogenic diet on HF and the operating mechanisms, administered daily for 8 weeks to mice, failed to show protection against HF [34]. However, alternate-day ketogenic diet feeding did exert potent cardioprotective effects against HF, while maintaining hepatic ketogenesis, effected via every other day but not a continuous (daily) ketogenic diet, deemed to account for this benefit. 

Thus, one should exert caution in implementing continuous ketogenic diet feeding in the treatment of HF; alternate-day ketogenic diet feeding seems to be a better alternative approach to the avail of its cardioprotective effects for HF as it maintains lipid homeostasis and preserves the capacity of hepatic ketogenesis, which is not the case with a continuous ketogenic diet [34]. 

Higher ketone body availability has been associated with beneficial CV effects, attributable to better cardiac energetics and decreased oxygen consumption [35]. Thus, a ketogenic diet has the capacity to both manage and avert CVD. Nevertheless, the ketogenic diet has certain untoward effects that could negate the favorable CV effects, such as hyperlipidemia with an increase of triglycerides and low-density lipoprotein (LDL) cholesterol concentrations in the blood (see discussion below). Furthermore, poor diet compliance and not knowing of other possible chronic effects may also limit the wider application of the ketogenic diet.

### 3.2. Intermittent Fasting

Intermittent fasting has been found to have favorable effects on HF [36]. The potential molecular mechanisms of these beneficial effects may involve liver ketone bodies with evidence indicating that intermittent fasting favors a metabolic shift in organs with high metabolisms, such as the heart, which augments the usage of ketone bodies while fasting [36]. Furthermore, ketone bodies are engaged in the signaling pathways that regulate the expression of genes participating in protection against oxidative stress and in metabolism. Various molecular agents, such as adenosine monophosphate-activated protein kinase (AMPK), sirtuins, fibroblast growth factor 21 (FGF21), nuclear factor erythroid 2-related factor 2 (Nrf2), and peroxisome proliferator-activated receptor, seem to participate. Additionally, intermittent fasting seems to preserve the circadian rhythm, which is important for organs which are very active in terms of metabolism. 

Ketone body β-OHB and FGF21 have been suggested to moderate systemic metabolic reaction to fasting. Indeed, findings of a recent experimental animal study indicate that fasting-induced β-OHB and circulating FGF21 in coordination control gene expression response to oxidative stress in the heart [37]. 

An animal study indicated that intermittent fasting commenced before or after a myocardial infarction (MI) in rats could reduce myocyte hypertrophy and left ventricular (LV) dilation [38]. Myocardial fibrosis and fetal gene expression were not affected by feeding regimens. The benefit was more evident when intermittent fasting began before rather than after the MI. Importantly, there is a paucity of human data in this regard. 

### 3.3. Ketone Bodies in Heart Failure 

Patients with HF may have increased cardiac use of ketones, but it is not clear whether the driver for consumption reflects greater accumulation and, therefore, higher myocardial availability of ketone bodies or greater myocardial avidity and preference for ketone bodies or both [39,40]. As mentioned, in patients with HF, ketone body plasma levels are increased and the failing heart redirects energy metabolism toward augmented use of ketone bodies at progressively higher rates, suggesting an adaptive response to stress [9,41]. A positive correlation has been noted between the enhanced energy metabolism of cardiomyocytes and the concentrations of β-OHB acid and acetone. Thus, as more ketone bodies become available, protection against HF is enhanced.

A prospective population-based cohort study investigated the link between plasma β-OHB and the risk of HF in 6134 individuals followed for a median of 8.2 years, whereby 227 persons were diagnosed with HF, of whom 137 developed HF with reduced LV ejection fraction (HFrEF) and 90 were diagnosed with HF with preserved ejection fraction (HFpEF) [42]. Cox regression analyses disclosed a significant association of elevated β-OHB blood levels with incident HF (hazard ratio-HR per 1 standard deviation -SD increase, 1.40; *p* < 0.001), which was mostly ascribable to HFrEF. In females, the HR for HFrEF per 1 SD increase in β-OHB was 1.73 (*p* = 0.005) in an analysis that was adjusted for several confounders (e.g., age, body mass index-BMI, type 2 diabetes, hypertension, MI, smoking, alcohol consumption, total cholesterol, HDL-cholesterol, triglycerides, glucose, renal function). In men, in the same fully adjusted analysis, the HR was 1.14 (*p* = 0.36; *p* < 0.01 for sex interaction). In N-terminal pro-brain natriuretic peptide (NT-proBNP)-stratified analysis, the age-adjusted link with HF was significant in women with higher NT-proBNP levels (*p* = 0.008). The authors concluded that high plasma concentrations of β-OHB reflect an increased risk of HFrEF, particularly in women. Such findings indirectly suggest that ketone bodies are recruited as a rescue fuel. 

In a similar context, a recent study disclosed that the ketone body levels were significantly linked with the B-type natriuretic peptide (BNP) level (*p* = 0.003) but not with hemodynamic parameters, such as the LV end-diastolic pressure or volume levels [43]. The authors suggested that these results indicate that increased blood concentrations of ketone bodies are more robustly triggered by the elevation of BNP than by hemodynamic worsening with BNP-inducing ketone body production for use as an alternate fuel source in the failing heart.

The failing heart encounters an energy shortage, mainly due to a reduction in mitochondrial oxidative ability [44]. This is counterbalanced in part by a rise in ATP generation from glycolysis. The respective participation of the various fuels in mitochondrial ATP generation is also adjusted, consisting of a reduction in glucose and amino acid oxidation and an enhanced ketone body oxidation. The oxidation of fatty acids by the myocardium is enhanced or decreased, according to the type of HF. Specifically, in HF encountered in diabetes and obesity, heart muscle fatty acid oxidation is enhanced, whereas, in HF that occurs in hypertension or ischemia, fatty acid oxidation in the myocardium diminishes. All these energy metabolic alterations end up in the failing heart being rendered less efficacious (i.e., a reduction in cardiac work/O_2_ consumption). The alterations that take place in the failing heart regarding glycolysis and mitochondrial oxidative metabolism are ascribed to both transcriptional modifications in principal enzymes taking part in these metabolic pathways, as well as changes in nicotinamide adenine dinucleotide (NAD) redox state, i.e., NAD oxidized (NAD^+^) and NAD reduced (NADH) concentrations and metabolite signaling that participate in the posttranslational epigenetic alterations in the regulation of expression of genes encoding metabolic enzymes involved in energy production. Changes in the metabolism of glucose, besides flux via glycolysis or glucose oxidation, are also responsible for the pathophysiology of HF. Importantly, targeting the energy metabolic pathways for pharmacological purposes aims at ameliorating the efficiency of the heart, reducing the energy shortage, and enhancing systolic/diastolic function of the failing heart. 

A posthoc analysis of 79 patients with acute HF participating in the EMPA-RESPONSE-AHF study, which comparatively assessed treatment with a sodium-dependent glucose-cotransporter protein 2 (SGLT-2) inhibitor, empagliflozin, for one month, with placebo therapy in patients with acute HF, showed that ketone body levels in the circulation, and particularly acetone, were significantly raised during an event of acute decompensated HF versus the period following stabilization [45]. Therapy with empagliflozin did not have an effect on ketone body levels in acute HF patients. Specifically, in this trial, plasma levels of ketone bodies (acetone, β-OHB, and acetoacetate) were measured at baseline (median 251 µmol/L) and at 5 different time points [45]. Ketone body levels gradually decreased to 202 µmol/L at 1 month (*p* = 0.041). In particular, acetone decreased from 60 µmol/L at baseline to 30 µmol/L (*p* < 0.001), whereas β-OHB and acetoacetate remained stable over time. Furthermore, higher acetone concentrations were correlated with higher NT-proBNP levels (r = 0.234; *p* = 0.039). Circulating ketone bodies did not differ between patients treated with empagliflozin or those receiving a placebo. Univariate analysis indicated that higher acetone levels at baseline conferred a greater risk of the composite endpoint (in-hospital worsening HF, HF rehospitalizations, and all-cause mortality) after 1 month. However, after adjustment for age and gender, acetone was not an independent predictor any longer for the combined end-point. The authors concluded that ketone body levels, and acetone in particular, were significantly elevated during an episode of acute decompensated HF compared with after stabilization. Empagliflozin did not influence ketone body levels in patients with acute HF. Thus, the fall in acetone might reflect effective decongestion rather than any particular effects of empagliflozin.

In summary, in patients with HF, ketone body production and concentrations are increased and the failing heart redirects energy metabolism toward the augmented use of ketone bodies as an alternate or rescue fuel source at progressively higher rates, suggesting an adaptive response to stress; as more ketone bodies become available, protection against HF is enhanced. 

### 3.4. Ketone Bodies as Biomarkers 

As mentioned, in patients with congestive HF, circulating ketone bodies are usually elevated, and their levels increase with worsening systolic myocardial function, commensurate with neurohormonal activation and with increasing atrial pressures and with the degree of venous congestion, partly ascribable to elevated FFA mobilization in response to heightened neurohormonal stimulation [9,46,47]. Acetone can be measured reliably in exhaled breath, acetoacetate in urine, and β-OHB in urine or via a finger-stick method by using portable devices, which renders them useful for routine clinical practice [48,49,50]. Indeed, much higher levels of exhaled acetone have been measured in patients with HF than in controls, and they correlate with circulating ketone bodies, while these levels are higher with worsening New York Heart Association (NYHA) class, the magnitude of peripheral edema, and the degree of left and right ventricular systolic dysfunction, and correlate with higher blood levels of natriuretic peptides. Importantly, in patients hospitalized with worsened HF having initially high concentrations of exhaled acetone, monitoring these levels as they decline, one can follow-up treatment efficacy and patient response to acute HF therapies, including their response to diuretics [51]. 

Thus, ketone bodies may constitute useful biomarkers of the severity of HF and its prognosis [40,46,47].

### 3.5. Atherosclerotic Heart Disease/Coronary Artery Calcification

Experimental studies have indicated that daily nutritional supplementation of β-OHB can attenuate atherosclerosis in mice by inhibiting the activation of the NOD-like receptor family, pyrin domain-containing protein 3 (NLRP3) inflammasome, which stimulates the elevation of M1 macrophage percentage and the suppression of cholesterol efflux [52]. 

Cross-sectional and longitudinal trials were performed in adults without DM or CVD to investigate the link between fasting ketonuria and coronary artery calcification (CAC) and its progression. Of 144,346 subjects included in these studies, 12.3% had CAC scores >0 at baseline. In general, increased fasting ketonuria was linked with a lower occurrence of CAC compared with no ketonuria [53]. Multivariable-adjusted odds ratios (ORs) for the prevalence of CAC by contrasting ketonuria levels 1 and ≥2 with no ketonuria were 0.94 and 0.82, respectively. The link was not different among clinically relevant subpopulations. Ketonuria was linked with decreased progression of CAC during follow-up (the multivariate-adjusted ratio of rates of ketonuria progression ≥2 compared with no ketonuria was 0.976). The authors concluded that an inverse link was found between ketonuria during fasting and subclinical atherosclerosis of the coronaries, in both occurrence and progression, suggesting a possible protective effect of augmented ketone body production in CVD that needs additional exploration. 

### 3.6. Myocardial Infarction

Higher circulating ketone bodies (β-OHB, acetoacetate, and acetone) at 24 h compared to baseline levels (at presentation) have been found in 369 patients hospitalized with ST-elevation myocardial infarction (STEMI), linked with functional outcomes following STEMI, which indicates a possible effect of ketone metabolism responding to myocardial ischemia [54]. Levels were still elevated after 4 months. Increased ketone body levels at 24 h were independently linked with bigger myocardial infarct (MI) size (*p* = 0.016) and lower LV ejection fraction (LVEF) (*p* = 0.012).

### 3.7. Cardiac Endothelial Cells

Cardiac endothelial cells have been reported to be able to oxidize ketone bodies with the use of succinyl-CoA:3-oxoacid-CoA transferase and this promotes cell proliferation, migration, and vessel sprouting [33]. An increase in ketone body concentrations by a high-fat, low-carbohydrate ketogenic diet was shown to increase endothelial cell proliferation in the hearts of mice temporarily. Interestingly, in a mouse model of cardiac hypertrophy, the ketogenic diet averted blood vessel rarefication, suggesting a possible benefit of dietary modification in CV disease.

### 3.8. Cardiac Hypertrophy

There is experimental evidence that the hypertrophied heart also turns to ketone bodies as an important energy fuel for oxidative ATP generation [55]. This shift to ketone body oxidation in the hypertrophied heart occurs as an early adjusted response to preserve sufficient fuel stock for oxidative ATP generation because of decreased oxidation of fatty acids, which constitute the main substrate for the physiologic heart. It seems that the hypertrophied and failing heart goes through gene regulatory reprogramming to augment the capacity for delivery and oxidation of ketone bodies in the heart. In particular, the expression of key enzymes in the ketone body oxidation pathway, such as the β-hydroxybutyrate dehydrogenase 1 (Bdh1) and the putative cellular ketone body transporter *Slc16a7*, is increased and upregulated in cardiac hypertrophy and HF [55]. 

In summary, there is an inverse link between higher ketone body levels and subclinical occurrence and progression of coronary atherosclerosis, pointing to a possible protective effect of augmented ketone body production in CVD. Also, higher circulating ketone bodies in patients with acute MI have been linked with functional outcomes in these patients, indicating a possible effect of ketone metabolism responding to myocardial ischemia. Cardiac endothelial cells have also been reported to be able to oxidize ketone bodies promoting cell proliferation, migration, and vessel sprouting. Finally, the hypertrophied heart also turns to ketone bodies as an important energy fuel for oxidative ATP generation due to a decrease in the oxidation of fatty acids, which constitute the main substrate for the physiologic heart. 

## 4. Hypertension 

Ketone supplements at low doses have been suggested as an effective antihypertensive treatment that reduces total peripheral resistance with very few untoward effects [56]. However, evidence about the antihypertensive effect of ketogenic diets remains ambiguous, especially in view of their possible side-effects, such as dyslipidemia, which might burden the hypertensive phenotype. Nevertheless, more recent evidence indicates that the most plentiful ketone body, β-hydroxybutyrate, can have favorable actions on endothelial and vascular health; thus, it is hoped that ketone bodies can be utilized as an effective antihypertensive approach. 

A prospective, double-blind, placebo-controlled, parallel-group single-center trial comprising 72 patients with HF (LVEF 39.0 ± 8.2%) randomly allocated (2:1) to the SGLT-2 inhibitor empagliflozin 10 mg orally qd or to placebo, who finished the study, showed that ketone body levels as represented by beta-hydroxybutyrate (β-OHB) concentrations rose after therapy with empagliflozin [57]. This increase produced a diminution of the favorable effects of empagliflozin on blood pressure and vascular parameters.

### Vascular Calcification

A recent trial showed that β-OHB supplementation inhibited vascular calcification in chronic renal disease (CKD) via modulation of the histone deacetylase (HDAC) 9-dependent nuclear factor kappa-light-chain-enhancer of activated B cells (*NF*-*κB*) signaling pathway [58]. Furthermore, a critical mechanistic effect of HDAC9 was shown in vascular calcification under CKD circumstances. The authors concluded that nutritional approaches or pharmacological strategies to raise β-OHB concentrations could be promising therapeutic goals to aim at HDAC9 for the management of vascular calcification in CKD. 

In summary, the antihypertensive efficacy of ketogenic diets is inconclusive; it is possible that side effects associated with ketogenic diets (e.g., dyslipidemia) aggravate the hypertensive phenotype. Nevertheless, ketone supplements warrant investigation as low-dose antihypertensive therapy that decreases total peripheral resistance with minimal side effects. Finally, strategies to raise β-OHB levels appear promising for the management of vascular calcification in CKD. 

## 5. Atrial Fibrillation

In addition to hypertension, HF, lung disease, and CAD which are the probable risk factors for atrial fibrillation (AF), the underlying molecular pathology for AF remains mostly unspecified [59]. The regression of the mature myocardial cells to fetal phenotype, compromised ketone body metabolism, impaired mitochondrial function and the cellular influence of ROS constitute major underlying biochemical actions linked with the molecular pathology of AF. Elevated concentrations of ketone bodies, mainly β-OHB and ketogenic amino acids, have been detected in persistent AF [59,60]. On the other hand, increased β-OHB levels and sirtuin 7 (SIRT7) expression, decreased mitochondrial biogenesis, and increased cardiac fibrosis were detected in human AF heart tissues; these alterations have been known to promote cardiac inflammation, hypertrophy, and fibrosis, all known to enhance atrial arrhythmogenesis [61,62,63]. 

In summary, persistent AF has been associated with higher levels of ketone bodies, possibly indicating a metabolic adaptation to this common arrhythmia in susceptible individuals. 

## 6. Diabetic Heart

Ketone bodies constitute great fuel (“super fuel”) and generate energy more effectively than fatty acids and glucose [28,64]. The EMPA-REG Outcome (Cardiovascular Outcome Event Trial in Type 2 DM Patients) trial with the use of an inhibitor of sodium-glucose cotransporter-2 (SGLT2i) (empagliflozin) indicates that utilizing ketone bodies in lieu of fatty acids could enhance cardiac efficiency in patients with diabetes mellitus (DM) [65]. The concentrations of ketone bodies in the circulation are high in DM due to enhanced hepatic ketogenesis; the cardiac supply of ketone bodies is also augmented in patients with vs. without DM; whereas the uptake of carbohydrates is decreased [66]. Thus, ketone bodies are employed as an energy fuel partially substituting glucose in the human diabetic heart. However, despite the high levels and enhanced uptake of ketone bodies in the heart, the diabetic heart is inefficient energy-wise, probably because of decreased use of ketone bodies via ketolysis in the diabetic heart [64,67,68]. The reason is not entirely clear for such an impaired ketolysis in the diabetic heart; reduced levels of ketolytic enzymes in both type 1 DM and type 2 DM (T2D) hearts indicate that compromised ketolysis could potentially explain this energy inefficacy in the diabetic heart [64]. It seems that elevated glucose levels reduce cardiac ketolytic activity and capacity via various mechanisms and point to a possible crosstalk between glucose and ketone body metabolism in the diabetic heart muscle. The hypothesis that has been put forth relates to enhanced transcription and function of ketogenesis enzymes in the heart that slow down ketolytic enzymes in the diabetic heart, which reduces myocardial energy efficiency [64,67]. In type 1 DM, the heart demonstrates increased upregulation of ketogenesis in comparison with the heart in type 2 DM due to the absence of insulin, which blocks ketogenesis enzymes [67]. 

### Insulin Resistance 

Heart failure often coexists with insulin resistance, and the incidence of HF in patients with T2D is significantly higher [69]. On the other hand, insulin resistance contributes to the upregulation of hepatic ketogenesis. In addition to ketone bodies, branched-chain amino acids (BCAA) (leucine, isoleucine, and valine) are essential amino acids which have critical roles in protein synthesis and energy metabolism in the body constituting another potential energy source for the heart. In T2D and a failing heart, metabolic flexibility is impaired; glucose uptakes and oxidation and fatty acid oxidation are impeded, and they rely more on alternative energy sources such as ketone bodies and BCAAs [70]. In the heart, there is a strong correlation between impaired BCAA oxidation and contractile dysfunction in HF [71]. BCAAs have been related to CVD but also play an important role in insulin resistance and T2D [72]. Elevated BCAA is a manifestation of insulin resistance [73]. The mechanism of BCAA overload causing insulin resistance may be the activation of the protein kinase mTOR (mammalian or mechanistic target of rapamycin), and the increase of acylcarnitine; mTOR is considered to be the main signal of crosstalk between BCAA and insulin [74]. Studies have found that higher BCAA levels are related to CVD [72]. On the other hand, lowering the BCAA in the diet will increase energy expenditure and improve insulin sensitivity. Furthermore, SGLT2 inhibitors can reduce plasma glucose and insulin levels and show significant improvement in insulin resistance and insulin secretion. The multiple roles of BCAA in the emergence of insulin resistance promise to be important and to lead to the development of novel effective T2D therapies [72]. 

In summary, utilizing ketone bodies in lieu of fatty acids as an energy fuel partly substituting for glucose could enhance cardiac efficiency in patients with diabetes. However, despite the increased levels and enhanced uptake of ketone bodies in the heart, the diabetic heart is still inefficient energy-wise, partly due to the lower use of ketone bodies via impaired ketolysis or an imbalance between ketogenesis and ketolysis enzymes in the diabetic heart. Importantly, HF often coexists with insulin resistance, which contributes to the upregulation of hepatic ketogenesis. Patients with T2D and a failing heart have impaired metabolic flexibility and they rely more on alternative energy sources such as ketone bodies and BCAAs, both related to CVD. Furthermore, impaired BCAA oxidation incurs contractile dysfunction; in addition, elevated BCAAs reflect insulin resistance. Finally, SGLT2 inhibitors can reduce plasma glucose and insulin levels and significantly ameliorate insulin resistance and insulin secretion. 

## 7. Sodium-Glucose Cotransporter 2 (SGLT-2) Inhibitors

Sodium-glucose cotransporter 2 (SGLT2) inhibitors have been demonstrated to elevate ketone bodies in patients with T2D [75]. A recent study provided data indicating that the dapagliflozin-induced elevation in plasma ketone bodies was steered by the combined effect of FFA mobilization from adipose tissue and redirection of liver FFA toward β-oxidation [76]. 

Canagliflozin treatment has been shown to increase plasma FFA and β-OHB irrespective of background antihyperglycemic treatment [77]. It seems that a constitutive metabolic plan including increased lipolysis may be advantageous in tarrying or avoiding hospitalization for HF; an additional activation of lipolysis by canagliflozin may strengthen this influence. 

In a study of targeted metabolomics in a randomized controlled trial (RCT) of SGLT-2 inhibitors (SGLT-2i) in HFrEF (Dapagliflozin Effects on Biomarkers, Symptoms, and Functional Status in Patients With HF With Reduced Ejection Fraction—DEFINE-HF), effects were observed of dapagliflozin on principal metabolic pathways, backing a role for modified ketone and fatty acid biology with SGLT-2i in patients with HFrEF [78]. Only expected levels of ketosis were noted. Specifically, among the 234 study participants (mean age 62.0 ± 11.1 years, 25% females, 38% Black, mean LVEF 27 ± 8%), dapagliflozin vs. placebo raised ketone-related and short-chain and medium-chain acylcarnitine main components analysis-defined metabolite clusters (baseline *p* = 0.01, false discovery rate-corrected *p* = 0.08 for both clusters). Nevertheless, ketosis (β-OHB concentrations >500 μmol/L) was scarcely observed (3 [2.5%] in the dapagliflozin arm vs. 1 [0.9%] in the placebo arm) and supranormal concentrations were not encountered. Elevations in long-chain acylcarnitine, long-chain dicarboxylacylcarnitine, and aromatic amino acid metabolite clusters were linked with impaired quality of life and higher NT-proBNP concentrations, without interaction noted by the therapy group. 

Finally, the outcome of a meta-analysis of 72 trials reporting information on diabetic ketoacidosis (DKA) in patients with T2D receiving SGLT-2 inhibitors, of which 9 trials observed at least one case of ketoacidosis, provided reassurance that, when these drugs are appropriately prescribed, there is negligible risk of DKA [79]. In keeping with these results, another more recent meta-analysis of 39 RCTs, involving 60,580 patients, reported 85 DKA events (SGLT2 inhibitors: 62 events or 0.18% vs. control: 23 events or 0.09%; Peto odds ratio—OR 2.13, I[2] = 8%; risk difference-RD 1.7 more events per 1000 over 5 years; high-quality evidence) [80]. Results did not differ by sensitivity analyses. Subgroup analyses indicated a larger relative effect among older patients (aged ≥60 years) and those with longer use of SGLT2 inhibitors (>52 weeks). 

Thus, under situations of mild, persistent hyperketonemia, such as those that develop during therapy with SGLT2 inhibitors, β-OHB is utilized by the heart and oxidized in lieu of FFA [65]. Ketone body selection as a fuel ameliorates the transduction of oxygen consumption into work efficiency at the level of the mitochondria. Furthermore, the hemoconcentration that often ensues after glycosuria from SGLT2 inhibition facilitates the release of oxygen to the tissues, thus conferring a robust synergistic effect on the shift of the metabolic substrate. 

A variety of mechanisms have been proposed to clarify the cardioprotective effect of SGLT2 inhibitors in patients with DM and/or HF [64]. One hypothesis is that gene expression occurs in the diabetic heart together with catalytic regulatory reprogramming that diminishes the intrinsic capacity for ketone body oxidation. Nevertheless, it remains unknown whether reduced gene expression in ketone body catabolism is the cause or effect of DM, and if increasing ketone body usage in the diabetic heart would also increase ATP generation. The EMPA-REG OUTCOME trial conducted in patients with T2D and increased CV risk has documented lower CV mortality in patients receiving empagliflozin [81]. Furthermore, the drug raised circulating ketone body concentrations in these patients, and it has been suggested that enhanced cardiac ketone oxidation accounted for the favorable effects in this study [65]. Also, another recent trial reported that T2D patients with diabetic ketosis had a lower total death rate versus those hyperglycemic patients with no ketosis, indicating that ketosis may likely be protective in DM representing a compensatory mechanism rather than an untoward effect in patients with hyperglycemic episodes [82]. 

In summary, SGLT2 inhibitors have been demonstrated to elevate ketone bodies in patients with T2D, which are utilized by the heart and oxidized in lieu of FFAs. A variety of mechanisms have been proposed to explain the cardioprotective and life-prolonging effects of SGLT2 inhibitors in patients with DM and/or HF. It has been suggested that enhanced cardiac ketone oxidation might account for these favorable effects and ketosis may be protective in DM representing a compensatory mechanism rather than an untoward effect in patients with hyperglycemic episodes. 

## 8. Ketone Bodies and the Heart/A Double-Edged Sword

The heart has metabolic flexibility. Under normal circumstances, it mostly utilizes lipids and glucose for the generation of energy [24]. In uncontrolled diabetes, the heart shifts its metabolism towards the utilization of lipids predominantly, which, however, in the long term may turn out to be deleterious to LV function. Furthermore, diabetes is characterized by high blood levels of ketones which are increasingly utilized for energy production. The current investigation explores whether exogenous ketone body administration might be of benefit in the management of CV disease, as it is still not clear if augmented utilization of cardiac ketones is advantageous or deleterious to cardiac performance and function. Lipid-induced cardiac dysfunction has been ascribed to the disassembly of the endosomal proton pump (the vacuolar-type H+-ATPase or v-ATPase) occurring first, ensued by endosomal de-acidification/dysfunction. The de-acidified endosomes can no more function as a storehouse for lipid transporter CD36, which subsequently translocates to the sarcolemma where it induces lipid aggregation, insulin resistance, and impaired systolic function. Lipid-induced v-ATPase disassembly is counter-balanced by the accumulation and stock of specific amino acids. A study examined the influence of ketone bodies on v-ATPase assembly status and control of lipid metabolism in rodent/human myocardial cells [24]. Exposure to β-OHB triggered v-ATPase disassembly and the ensuing events led to systolic dysfunction and insulin resistance, akin to a state of excessive lipid supply. Adding acetoacetate did not trigger v-ATPase dysfunction. The adverse effects of 3β-OHB could be avoided by adding specific amino acids, which could potentially be a therapeutic option.

A recent small randomized controlled trial (RCT) with a crossover design alleviated concerns about whether acute ketosis might have an adverse effect on plasma lipid profile by studying 18 adults (12 men, 6 women) with prediabetes, who, following an overnight fast, ingested a single dose of ketone monoester β-hydroxybutyrate (KEβHB) drink or placebo drink [83]. A considerable increase of blood β-OHB from 0.2 to 3.5 mmol/L (*p*< 0.001) was noted within 30 min. Acute ketosis led to considerably reduced areas under the curve for remnant cholesterol (*p* = 0.022) and triglycerides (*p* = 0.022), with no important changes in total cholesterol, LDL cholesterol, HDL cholesterol, and the triglycerides to HDL cholesterol ratio. The differences in triglycerides and remnant cholesterol were statistically significant in persons with high, but not low, regular saturated fat ingestion. The authors alleged that these findings pave the way for exploring if exogenous supplementation of ketone bodies diminishes CV risk through their effects on triglyceride-rich lipoproteins in high-risk patient groups. 

As detailed above, β-OHB has been considered to be involved in the energy metabolism of the failing heart as an alternate “fuel source”. A failing heart can increase utilization of β-OHB, and this functions as a “fuel switch” that has been shown to be an adaptable reaction to stress with worsening of HF in both patients with and without diabetes [17]. Besides functioning as an alternate “fuel”, β-OHB is a signaling molecule that functions as an endogenous histone deacetylase (HDAC) inhibitor. It is able to enhance histone acetylation or lysine acetylation of other signaling molecules. Also, β-OHB has been demonstrated to reduce the generation of ROS and instigate autophagy. Furthermore, β-OHB functions as an inhibitor of the NLR family pyrin domain-containing protein 3 (Nlrp3) inflammasome and decreases Nlrp3-mediated inflammatory reactions. In addition, β-OHB can participate in transcriptional or post-translational control of several genes’ expression. Raising β-OHB concentrations before inducing ischemia/reperfusion injury leads to a decreased infarct size in animal experiments, probably ascribable to the signaling operation of β-OHB besides its role in providing energy. The clinical efficacy of SGLT2 inhibitors in HF and CVD may be partly explained by their effect of raising the generation of β-OHB. Nevertheless, in spite of all the favorable actions of β-OHB, some trials have reported deleterious actions of prolonged use of β-OHB. Also, not all methods of raising β-OHB concentrations in the myocardium are similarly efficacious in managing HF. We are still in dire need of studies to determine the best timing and treatment approaches for the administration of β-OHB to manage CVD. 

Importantly, the timing, mode of delivery, and duration of exposure to β-OHB need to be refined as it was found that lengthened subjection to β-OHB had a deleterious influence on myocardial cells by changing glucose uptake and enhancing the generation of ROS by blocking the activation of AMPK/p38 MAPK signaling pathway [84]. This was also corroborated by other trials which showed that a lengthened exposure to β-OHB effected via profound fasting or frequent exogenous administration can block mitochondrial biogenesis and produce atrial fibrosis by activating Sirtuin 7 (Sirt7) transcription via blocking histone deacetylases 2 (HDAC2) [61]. 

Thus, one should underscore the significance of the timing and dosages of β-OHB administered in various CVD models, i.e., diabetic cardiomyopathy, ischemia/reperfusion (I/R) injury, or pressure-overload. Hence, additional data and trials are required in order to determine which is the optimal way of administration of ketone bodies as possible therapeutic means for several CV diseases [17]. 

### Lean Mass Hyper-Responders

A curious observation concerns lean individuals with low triglycerides and high HDL-cholesterol who may develop a marked increase in plasma LDL-cholesterol when consuming a very low-carbohydrate ketogenic diet, a phenomenon, which has been termed the lean mass hyper-responder (LMHR) phenotype [85,86]. This phenotype was defined as LDL-cholesterol ≥200 mg/dL, HDL-cholesterol ≥80 mg/dL, and triglycerides ≤70 mg/dL. Despite the elevated HDL-cholesterol in this cluster and its attendant protective role in atherosclerotic CVD, the CV safety of the ketogenic diet promoting a significant rise of LDL cholesterol is put into question; especially in view of the firmly established atherosclerotic CV risk of apoB-containing lipoproteins, primary carriers of LDL-cholesterol [87]. 

In summary, it is still not entirely clear if augmented utilization of cardiac ketones is advantageous or deleterious to cardiac performance and function, the problem is the emergence of potential adverse effects with the administration of exogenous ketones, although it seems that such effects when concerning β-OHB could be avoided by supplementing specific amino acids, which could potentially be an added therapeutic option. Also, concerns of acute ketosis adversely affecting the plasma lipid profile have been alleviated by recent encouraging findings. Nevertheless, one should be vigilant about the potential deleterious actions of prolonged use of β-OHB. Also, not all methods of raising β-OHB concentrations in the myocardium are similarly efficacious in managing HF. We still need future studies to determine the best timing and treatment approaches for the administration of β-OHB to manage CVD. Finally, the curious observation of an LMHR phenotype concerning lean individuals with low triglycerides and high HDL-cholesterol who may develop a marked increase in plasma LDL-cholesterol when consuming a very low-carbohydrate ketogenic diet needs further investigation. 

## 9. Mitochondria and Oxidative Stress

Ketone bodies not only constitute a fuel source but also enhance resistance to oxidative and inflammatory stress, while there is a reduction in anabolic insulin-dependent energy consumption [32,88]. It has been proposed that ketone bodies act as a ligand to particular cellular targets and thus confer advantageous non-metabolic effects on organ functions. Another pathway has been proposed which is started by the initiation of oxidative stress in the mitochondria during enhanced ketolysis. Oxidative stress triggered by ketone body metabolism offers a long-term advantage as it instigates an adaptive (hormetic) response marked by the stimulation and mobilization of the master regulators of cell-protective mechanism, sirtuins, nuclear factor erythroid 2-related factor 2 (Nrf2), and AMP-activated kinase [32]. This leads to reduction or elimination of oxidative stress, by the upregulation of anti-oxidative and anti-inflammatory processes, ameliorated function and growth of the mitochondria, and better DNA repair and autophagy. In the heart, the adjustable and flexible response to increased ketolysis enhances resistance to injury after ischemic insults or to the cardiotoxic effects of drugs such as doxorubicin.

Most studies associate β-OHB with the attenuation of oxidative stress, as its administration inhibits the production of reactive oxygen species-ROS/superoxide, prevents lipid peroxidation and protein oxidation, raises the levels of antioxidant proteins, and ameliorates mitochondrial respiration and production of ATP [7].

Interestingly, the oxidation of fatty acids in the myocardium is augmented or reduced, depending upon the type of HF; in HF linked with diabetes and obesity, myocardial fatty acid oxidation is augmented, whereas in HF linked with hypertension or ischemia, cardiac fatty acid oxidation is reduced [44]. 

## 10. Cardiomyocyte Electrophysiology

A recent experimental study evaluated the actions of two commercially available β-OHB preparations, an enantiomer R β-OHB, and a racemic mixture ±β-OHB, on invoked pluripotent stem cell cardiomyocyte electrophysiology [89]. Cardiomyocytes were cultured in R β-OHB or racemic β-OHB for a minimum of 10 days after lactate selection. Flouvolt or Fluo-4 was employed to assess cardiomyocyte electrophysiology. It was revealed that both preparations augmented the optical potential amplitude. However, while R β-OHB lengthened the action potential duration, the racemic β-OHB decreased the action potential duration. Additionally, the racemic β-OHB raised the peak calcium transient, but the R β-OHB decreased the peak calcium transient. Co-culturing with glucose or fatty acids did not improve these actions, implying that β-OHB was not only just a fuel source. The authors suggested that the effect of β-OHB on pluripotent stem cell cardiomyocyte electrophysiology is most probably stereoselective, and there is a need to assess the role of exogenous β-OHB in healthy individuals and patients with CV disease.

## 11. Ketone Therapy 

Animal data suggest that the heart employs ketone bodies as a defense against metabolic stress and propose that approaches targeting an increase of ketone supply to the heart could turn out to be helpful in HF therapy [90]. A meta-analysis of 43 trials (N = 586) assessing exogenous ketones and blood glucose showed that acute intake of exogenous ketones can raise blood β-OHB levels and reduce blood glucose levels [91]. 

A recent animal study indicated that ketone bodies may ameliorate the status of diabetic cardiomyopathy by decreasing fatty acid metabolism, augmenting ketone body usage, and reducing endoplasmic reticulum inflammation and stress [92]. 

Another animal study indicated that chronic therapy with exogenous ketone bodies was beneficial to the failing heart as it suppressed the worsening of myocardial function in ketone-treated animals, suggesting that a chronic increase of ketone bodies may be a treatment alternative for HF [93]. The reason behind this beneficial effect of ketones in HF patients may relate to the fact that the failing heart depends more on ketone bodies as a fuel source than previously acknowledged; in addition, ketone bodies act as signaling molecules that can quell systemic and myocardial inflammation [94]. Therefore, it is likely that deliberately raising circulating ketones, which are easily and efficiently oxidized by myocardial cells and capable of furnishing adjunct fuel for the energy-deprived and energy-starved failing heart, may be advantageous as an ancillary therapy for HF [41,94]. Furthermore, ketone bodies may assist in the recovery of myocardial function by suppressing oxidative stress, inflammation, and myocardial remodeling [41]. 

In keeping with the above, a recent animal study explored the mechanism of possible cardioprotection conferred by a newly developed and clinically safe ketone ester [95]. A model of lipopolysaccharide (LPS)-induced sepsis was utilized in which an augmented inflammatory response and reaction can lead to the dysfunction of several organs. Oral intake of ketone ester for 3 days before LPS injections provided mice with significant protection against strong and extensive systemic inflammation in comparison to the vehicle-treated mice. Importantly, the ketone ester guarded mice against sepsis-induced impairment of myocardial function as well as kidney dysfunction and fibrosis. It also mitigated the sepsis-induced inflammation of the myocardium, liver, and kidney. Interestingly, these beneficial effects were independent of alterations in the enzymes taking part in the metabolism of ketones. The authors concluded that the use of an exogenous ketone body suppresses or improves the dysregulated systemic and organ inflammation and attendant organ dysfunction in a model of extensive inflammation, suggesting a protective anti-inflammatory effect of ketones in sepsis and/or other inflammatory reactions. 

As indicated above, ketone bodies function as signaling molecules that can suppress systemic and cardiac inflammation, and hence it makes sense to elevate circulating ketones, expecting a beneficial effect as an adjunct mode of treatment for HF. In animal experiments, genetic or dietary induction of ketosis, and also exogenous administration of ketone bodies, have been shown to cause, albeit inconsistently, improvements in cardiac structure and function [94]. In humans, little is known about the effects of exogenous ketone bodies on cardiac function. A single-arm study showed that within 30 min of inducing modest ketosis in healthy fasting persons after ketone ester ingestion, an increase in LVEF, tricuspid annular planar systolic excursion, left atrial contraction, heart rate, and systolic blood pressure and a decrease in systemic vascular resistance were observed, similar to several effects noted in the failing heart [78]. Another randomized crossover study examined the CV effects of elevated circulating plasma concentrations of 3-OHB in HF patients in small groups of chronic HFrEF patients (LVEF: 37 ± 3%) [96]. Infusion of 3-OHB raised circulating concentrations of plasma 3-OHB from 0.4 ± 0.3 to 3.3 ± 0.4 mM (*p* < 0.001). Cardiac output increased by 2.0 ± 0.2 L/min (*p* < 0.001) due to a rise in stroke volume of 20 ± 2 mL (*p* < 0.001) and a heart rate of 7 ± 2 bpm (*p* < 0.001). Also, LVEF increased by 8 ± 1% (*p* < 0.001) numerically. A dose-response association was observed with a considerable elevation of cardiac output (0.3 L/min) already at plasma-3-OHB concentrations of 0.7 mM (*p* < 0.001). Finally, 3-OHB raised oxygen consumption (MVO_2_) without affecting myocardial external energy efficiency. A similar response was elicited in age-matched volunteers. 

As also mentioned, it was recently demonstrated that β-OHB supplementation inhibited vascular calcification in CKD through regulation of the HDAC9-dependent *NF*-*κB* signaling pathway [58], indicating that nutritional intervention or pharmacological strategies to increase β-OHB concentrations could prove a promising therapeutic intervention that targets HDAC9 for the management of vascular calcification in CKD.

Finally, as also mentioned before, a word of caution needs to be uttered, as there remain unknown deleterious effects of ketones which can lead to cardiac fibrosis and strategies are direly needed for averting myocardial fibrosis in patients for whom ketogenic diets and/or ketone body therapies are medically necessary [61]. Thus, further data and studies are required in order to determine how to deliver ketone bodies as potential therapeutic means for several CV diseases [17]. 

Nevertheless, other data show that chronically raising ketone bodies in the circulation can decrease myocardial inflammation and consequent fibrosis and thus blunt the development of HF [97]. This protective effect of a chronic increase of circulating ketone bodies against the emergence of HF may be ascribed to the capacity of β-OHB to decrease inflammation. These advantageous actions of ketone bodies have been associated with decreased cardiac nucleotide-binding domain-like receptor protein 3 (NLRP3) inflammasome activation, indicating that ketone bodies may attenuate myocardial inflammation through this mechanism. 

### Role of Body Weight 

With regards to the role of body weight on the CV effects of a ketogenic diet, it is apparent that among the strategies over the recent years for weight loss, the ketogenic diet is effective with its established benefits as a reliable nutritional approach that has a solid physiological and biochemical basis and is able to induce effective weight loss along with improvement in several CV risk parameters [98]. Importantly, low-carbohydrate ketogenic diets effectively improve CV risk factors (blood glucose, weight, and lipids) in obese/overweight patients, especially those with T2D when compared with non-ketogenic diets [99]. Furthermore, there is a plethora of benefits of such a diet that go beyond the mere body weight/ adiposity reduction, as detailed herein, and also shown in real-world settings [100]. In this context, in normal-weight individuals, a non-calorie-restricted ketogenic diet can produce a modest reduction in body mass and percent fat and can maintain fat-free mass while it leads to increased use of fat as fuel [101]. Furthermore, ketones have a potential benefit in patients with CVD; particularly in patients with HF [102], they might offset senescent changes leading to apoptosis-induced myocardial atrophy and failure [103]; they may benefit patients with hypertension and endothelial dysfunction [104], they can enhance cardiac endothelial cell proliferation [33], upregulate anti-oxidative and anti-inflammatory activities, and they can finally improve mitochondrial function and growth, DNA repair, and autophagy [32]. In the heart, the adaptive response to increased ketolysis ameliorates resistance to damage after ischemic episodes or to cardiotoxic effects of chemotherapeutic agents, like doxorubicin [32].

In summary, in view of the promising and favorable effects of endogenous ketones recruited as an alternative fuel for the failing heart and other CV diseases, the current investigation explores whether exogenous ketone body administration might be of benefit in the management of patients with CVD. The benefits of ketogenic diets seem to extend to all patients regardless of body weight as these diets can be adjusted with regard to their caloric intake. However, there is still some concern about the safety of the ketogenic dietary approach as some trials have reported deleterious effects of prolonged use of β-OHB. Furthermore, not all methods of raising β-OHB or other ketone concentrations in the myocardium pertaining to the timing, mode of delivery, and duration of therapy are similarly efficacious or safe in managing HF and other CV diseases. We are still in dire need of further relevant studies to determine the best timing and treatment strategies.

## 12. Exercise 

It has been reported that regular exercise can raise serum ketone body (β-OHB) levels, and this action has been suggested as a crucial mechanism of exercise halting the emergence of atherosclerosis [105]. Specifically, an experimental study showed that 3 months of habitual exercise on a treadmill considerably reduced lipid aggregation and formation of foam cells in ApoE^−/−^ mice fed with a Western atherosclerotic diet [105]. This intervention increased β-OHB levels in the serum. Furthermore, β-OHB therapy in vivo or in vitro increased the protein concentrations of cholesterol transporters, comprising ATP-binding cassette transporters A1 and G1 (ABCA1, ABCG1), and scavenger receptor class B Type I (SR-BI), and was able to decrease lipid aggregation. Furthermore, it attenuated autophagy in macrophages and atherosclerotic plaques, which have a considerable action in the process of cholesterol efflux. This interesting finding whereby an elevation in serum β-OHB levels following regular exercise may be a significant mechanism that could inhibit the emergence of atherosclerosis might imply that those patients with atherosclerosis who are unable to exercise might benefit from supplemental β-OHB. 

## 13. Controversial Issues

A controversial issue relates to the lean mass super-responder phenotype promoted by a ketogenic diet, whereby LDL-cholesterol is markedly elevated, putting into question the CV safety of the ketogenic diet (see discussion above) [86]. 

Another issue relates to the possible adverse effects of ketone bodies on vascular function and structure, as β-OHB has been shown to attenuate the beneficial effects of empagliflozin on vascular function and blood pressure (BP) (although, overall, BP decreased and vascular function improved after treatment with empagliflozin), suggestive of early functional endothelial damage and vascular stiffness, increased after treatment with a ketogenic diet [106]. Similar results were reported in an animal study where the ketogenic diet impaired endothelium-dependent relaxation in mesenteric arteries and worsened hypertension in spontaneously hypertensive rats [107]. Furthermore, the ketogenic diet has been shown to induce an increase in inflammatory parameters and enhance oxidative stress through an increase in reactive oxygen species (ROS) [107,108]. 

Regarding the controversy on the effect of ketones on BP, a study by Pietschner et al. showed that empagliflozin led to an increase of fasting serum β-OHB in patients with stable HF compared to the placebo group with an attendant attenuated reduction of empagliflozin-induced 24 h systolic and diastolic BP [57]. On the other hand, Costa et al., who talk about the Janus face of ketone bodies in hypertension, indicate that the antihypertensive efficacy of ketogenic diets is inconclusive; it is possible that side effects associated with ketogenic diets (e.g., dyslipidemia) aggravate the hypertensive phenotype [56]. Nevertheless, they propose that ketone supplements warrant investigation as low-dose antihypertensive therapy that decreases total peripheral resistance with minimal adverse effects.

Other issues that might be considered controversial have already been discussed above (also see the Section 8) and relate to the mode of continuous (daily) vs. alternate-day feeding of a ketogenic diet, the inconclusive evidence of the antihypertensive efficacy of ketogenic diets, the emergence of potential adverse effects of exogenous ketone administration, particularly when it is prolonged, including a possible adverse effect on lipid profile, and finally, the curious observation of the LMHR phenotype (see relevant subsection). Thus, we are still in dire need of further studies on these issues. 

## 14. Conclusions

In situations that end up in exhausted glucose levels in the circulation, ketone bodies produced in the liver may constitute fuel substrates for the brain, while current evidence indicates that the heart, which utilizes mainly fatty acids as an alternative fuel source, can also avail itself from ketone bodies as a fuel source. Importantly, during pathophysiological conditions, such as HF and other CV conditions, alterations in metabolic processes that physiologically entail adequate production of energy from fatty acids and carbohydrates lead to a diminution in systolic cardiac function. Thus, it would be preferable that the failing heart trusts and depends more on ketone bodies as an energy fuel under these circumstances. Interestingly, it has been demonstrated that ketone bodies act as signaling molecules that can contain or curb systemic and myocardial inflammation. Hence, the suggestion that exogenous or endogenous means of supplying ketone bodies to circulation may be advantageous as an additive or ancillary therapy for HF and other CV diseases, such as post-MI. Therapeutic and dietary strategies to improve the delivery of ketone bodies to the heart are outlined in Table 1. Finally, one should note that under normal conditions, ketone body concentrations are low and should be cautious in considering high blood ketone concentrations as the new norm for patients with HF or other CVD, as such an aberration suggests situations of increased oxidative stress, dietary insufficiency of or inability to metabolize carbohydrates, mitochondrial dysfunction with its attendant ailments, a pro-inflammatory state, while one also needs to look out for the potentially deleterious effects of these compounds, including a dyslipidemic profile. 

## 15. Perspective

The heart, among other highly metabolic organs, increases the use of ketone bodies during fasting or periods of deficient food supply or ailments such as HF. Besides their role as energy substrates, ketone bodies participate in the signaling pathways that regulate the expression of genes involved in oxidative stress protection and metabolism. The increased production and use of ketone bodies as ancillary fuel during periods of shortage in food supply and low insulin levels cause oxidative stress in the mitochondria which initiates a cardioprotective response that allows myocardial cells to cope with higher oxidative stress and lower energy availability. In turn, this results in resolving oxidative stress, by the upregulation of anti-oxidative and anti-inflammatory activities, ameliorated mitochondrial function and growth, DNA repair, and autophagy. In the heart, the adaptive response to enhanced ketolysis improves resistance to damage after ischemic insults or to cardiotoxic actions of drugs. Importantly, SGLT2 inhibitors may also exert their cardioprotective action via increasing ketone body levels and ketolysis.

Chronic treatment with exogenous ketones seems to be of benefit to the failing heart and chronic ketone elevation may be a therapeutic option for HF and CVD in general. Indeed, increased availability of ketones is associated with beneficial CV effects, as they blunt the decline of systolic function in the failing heart, possibly explained by improved cardiac energetics and reduced oxygen use [93]. Furthermore, chronic treatment with SGLT-2 inhibitors, which decreases the severity of HF, also increases the ketone body supply to the heart. While ketogenic diets increase circulating ketone levels, a minimal benefit on cardiac function in HF has been noted, possibly due to the fact that these dietary regimens also markedly increase circulating fatty acids that may negate the ketone-conferred benefits. Recent studies, however, have suggested that the administration of ketone ester cocktails may improve cardiac function in HF [44]. Thus, ketone bodies have the potential to both treat and prevent CVD.

However, ketone body therapy has some adverse effects that could negate the beneficial CV properties. Of these, hyperlipidemia with an elevation of triglycerides and LDL cholesterol levels is very important. Furthermore, poor diet adherence and lack of knowledge regarding the long-term effects may also curtail the broader applicability of ketone therapy.

Future investigations may elucidate the mechanisms underlying such protective and/or untoward effects and find ways to harness this ancillary fuel for selective beneficial usage. One important aspect may have to do with ketone body concentrations in relation to their effects. At low concentrations, endogenously produced ketone bodies by implementing a ketogenic diet or exogenously supplemented ketone bodies (or their precursors) may prove beneficial to enhance endothelial function and relevant CV pathologies [109].

## Figures and Tables

**Table 1:** Therapeutic and dietary strategies to enhance the delivery of ketone bodies (KB) to the heart.

Intravenous infusion of KB raises circulating KB levels along with their ensuing supply to the heart
Ketone esters can be given orally whereby they are cleaved into ketone bodies in the gastro-intestinal tract and released into the circulation by the liver, increasing ketone body supply to the heart
The ketogenic diet (high-fat/low-carbohydrate diet) increases the supply of fatty acids to the liver with an attendant enhanced ketogenesis in the liver raising circulating fatty acid levels and their supply to the heart
Sodium-glucose co-transporter-2 (SGLT2) inhibitors increase glucose excretion in the urine and produce a fasting-like state with a resultant increase in lipolysis in the adipose tissue and delivery of fatty acids to the liver
Similar to a ketogenic diet, SGLT2 inhibitors increase ketogenesis in the liver, raising circulating ketone levels and ketone delivery and supply to the heart

## Data Availability

Data sharing not applicable to this article as no datasets were generated or analysed during the current study (review article).

## Abbreviations

AcAc = acetoacetate; acetyl-CoA = acetyl-coenzyme A; AF = atrial fibrillation; ATP = adenosine triphosphate; BNP = B-type natriuretic peptide; β-OHB = beta-hydroxybutyrate; CAC = coronary artery calcification; COVID-19 = coronavirus disease 2019; CV = cardiovascular; CVD = cardiovascular disease; DM = diabetes mellitus; FFA = free fatty acid(s); FGF21 = fibroblast growth factor-21; HF = heart. failure; HFpEF= heart failure with preserved ejection fraction; HFrEF = heart failure with reduced ejection fraction; 3-HMGC = 3-hydroxy 3-methylglutaryl-CoA; LDL = low-density lipoprotein; LPS = lipopolysaccharide; LV = left ventricular; LVEF = left ventricular ejection fraction; MCTs = monocarboxylate transporters; MI = myocardial infarction; NLRP3 = nucleotide-binding domain-like receptor protein 3; Nrf2 = nuclear factor erythroid 2-related factor 2; NYHA = New York Heart Association; ROS = reactive oxygen species; SARS-CoV-2 = severe acute respiratory syndrome *coronavirus 2*; SGLT-2 = sodium glucose cotransporter 2; STEMI = ST-elevation myocardial infarction; T2D = type 2 diabetes.
